# Genome integrity as a potential index of longevity in Ashkenazi Centenarian’s families

**DOI:** 10.1007/s11357-024-01178-0

**Published:** 2024-05-09

**Authors:** Mariana Andrawus, Gil Ben David, Ivana Terziyska, Lital Sharvit, Aviv Bergman, Nir Barzilai, Srilakshmi M. Raj, Diddahally R. Govindaraju, Gil Atzmon

**Affiliations:** 1 Faculty of Natural Sciences, University of Haifa, Haifa, Israel; 2 Sagol Department of Neurobiology, Faculty of Natural Sciences, University of Haifa, 199 Aba Khoushy Ave., 3498838 Mount Carmel, Haifa Israel; 3 Cornell University, Ithaca, NY 14853 USA; 4 Department of Systems and Computational Biology, Albert Einstein College of Medicine, Bronx, NY 10461 USA; 5 Departments of Medicine and Genetics, Albert Einstein College of Medicine, Bronx, NY 10461 USA; 6 Department of Genetics, Albert Einstein College of Medicine, Bronx, NY 10461 USA; 7 MCZ, Harvard University, Cambridge, MA 02138 USA

**Keywords:** Senescence, Copy number variants, Centenarians, Genome plasticity

## Abstract

The aging process, or senescence, is characterized by age-specific decline in physical and physiological function, and increased frailty and genomic changes, including mutation accumulation. However, the mechanisms through which changes in genomic architecture influence human longevity have remained obscure. Copy number variants (CNVs), an abundant class of genomic variants, offer unique opportunities for understanding age-related genomic changes. Here we report the spectrum of CNVs in a cohort of 670 Ashkenazi Jewish centenarians, their progeny, and unrelated controls. The average ages of these groups were 97.4 ± 2.8, 69.2 ± 9.2, and 66.5 ± 7.0 respectively. For the first time, we compared different size classes of CNVs, from 1 kB to 100 MB in size. Using a high-resolution custom Affymetrix array, targeting 44,639 genomic regions, we identified a total of 12,166, 22,188, and 10,285 CNVs in centenarians, their progeny, and control groups, respectively. Interestingly, the offspring group showed the highest number of unique CNVs, followed by control and centenarians. While both gains and losses were found in all three groups, centenarians showed a significantly higher average number of both total gains and losses relative to their controls (*p* < 0.0327, 0.0182, respectively). Moreover, centenarians showed a lower total length of genomic material lost, suggesting that they may maintain superior genomic integrity over time. We also observe a significance fold increase of CNVs among the offspring, implying greater genomic integrity and a putative mechanism for longevity preservation. Genomic regions that experienced loss or gains appear to be distributed across many sites in the genome and contain genes involved in DNA transcription, cellular transport, developmental pathways, and metabolic functions. Our findings suggest that the exceptional longevity observed in centenarians may be attributed to the prolonged maintenance of functionally important genes. These genes are intrinsic to specific genomic regions as well as to the overall integrity of the genomic architecture. Additionally, a strong association between longer CNVs and differential gene expression observed in this study supports the notion that genomic integrity could positively influence longevity.

**Supplementary Information:**

The online version contains supplementary material available at 10.1007/s11357-024-01178-0.

## Introduction

Biological aging process represents declining vitality and fertility along with increased frailty and vulnerability to diseases [1]. It is influenced by an interplay of genomic, epigenomic, and environmental factors during the life span of all organisms. A greater incidence of deleterious mutations, often leading to diseases, is associated with genomic damage, instability, and a shortened lifespan [2, 3]. In contrast, long-lived individuals tend to have fewer harmful germline mutations that manifest later in life, which also exhibit genetic robustness [4]. Exceptionally long-lived individuals (ELLI) have been shown to exhibit a slower aging rate, as measured by DNA methylation clocks, which serve as markers of aging [5]. Age-related genomic variation may occur randomly due to both genetic and environmental causes [6, 7]. These may also lead to random structural changes due to breaks in the chromosome strands, which are described as CNV [8].

Substantial variation exists in the frequency, rate of accumulation of mutations, and consequences of CNVacross various diseases, populations, and age groups. The manifold effects of loss or gain copy number changes have been shown to play a critical role in the initiation and progression of cancer, cognitive disorders, and diseases involving tandem repeat expansions [7, 9, 10] among other genomic diseases [9]. For instance, addition of genomic material through gene duplications could be advantageous as they could potentially influence vitality [11] and longevity (e.g., *SIRT2*, *FOXO3* [12]), as well as facilitate robustness against deleterious mutations through functional complementation [13].

Conversely, loss of CNVs may involve removal of entire genes and their regulatory regions which can lead to adverse health outcomes [10]. For instance, greater loss of large genomic regions (> 500 kB) has been shown to be associated with increased rate of mortality [14]. With age, CNVs accumulate differently between monozygotic twins [7], and structural variants such as translocations accumulate at greater frequencies even among healthy individuals, compared to newborns [15].

In contrast, chromosomal structural variants occur at lower frequencies among individuals 85 years or older (“oldest old”) relative to younger individuals [16]. In nematodes, long-lived strains (e.g., daf-2) have been found to accumulate fewer CNVs compared to their wild-type counterparts [17]. The latter studies [16, 17] suggest that although both gain and loss of genomic regions are inextricable aspects of the aging process, in very long-lived individuals, these processes appear to operate at a slower pace. In support of this, Stoeger et al. reported a direct relationship between transcript length and lifespan both in humans and in mice [18]. Therefore, from both a demographic and developmental standpoint, variations in the number of CNVs—whether gains or losses—could have either advantageous or deleterious effects on longevity [19, 20].

Previous studies have shown that Ashkenazi Jewish centenarians (AC) and their progeny maintain longer telomeric and sub-telomeric regions, and well-known genomic structural variants, compared to individuals with normal lifespan [21, 22]. Based on this observation, we proposed the following theories: (1) their telomere attrition rate may be slower, (2) the centenarian’s attrition rate is “normal” yet they began life with exceptionally long telomeres (as demonstrated by their offspring), (3) the centenarian's attrition rate is “normal,” however, at a certain age telomerase activity, regulated by HDL levels, becomes activated [23]. Such a perspective can be adapted to the offspring’s CNV excessive number. They also display enhanced cognitive function and favorable lipid profiles, both of which are indices of healthy aging [24, 25]. However, the impact of gains and losses in other genomic regions on the longevity of ACs remains unexplored. The unique demographic genetic features of the Ashkenazi Jewish population offer an unparalleled opportunity to investigate the influence of CNVs in the aging process. In this study, we address three major questions utilizing a cohort of Ashkenazi Jewish centenarians, which comprises 287 centenarians, 153 of their offspring, and 230 controls. First, does the spectrum of CNVs differ among these three groups within the AC cohort? Second, do these groups possess unique CNVs? Lastly, we explore whether some of these unique CNVs show plasticity and a functional relationship to various components of aging.

## Results

### CNV frequency and distribution by group

**Fig. 1:**
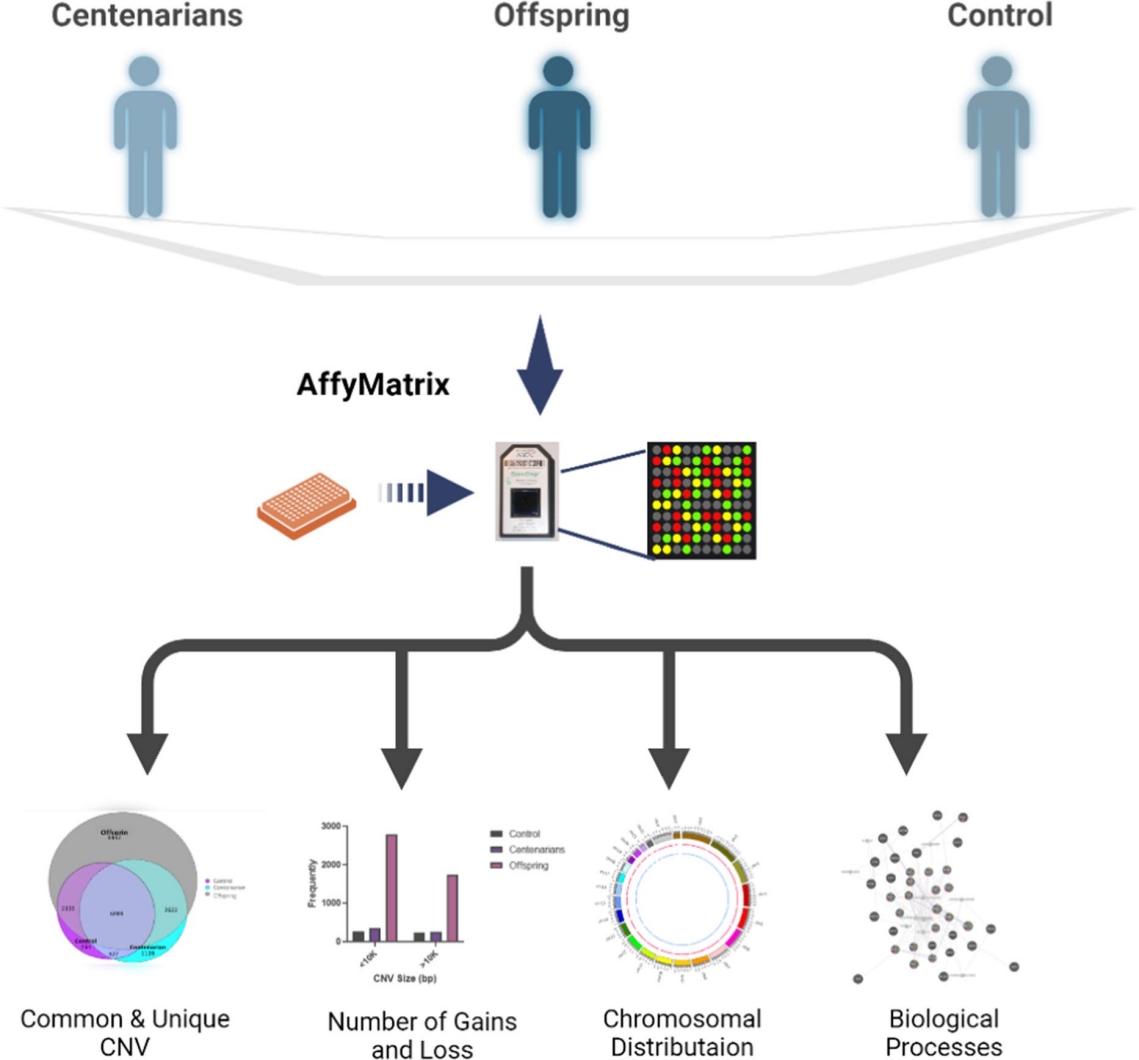
schematic overview of project’s steps

**Fig. 2:**
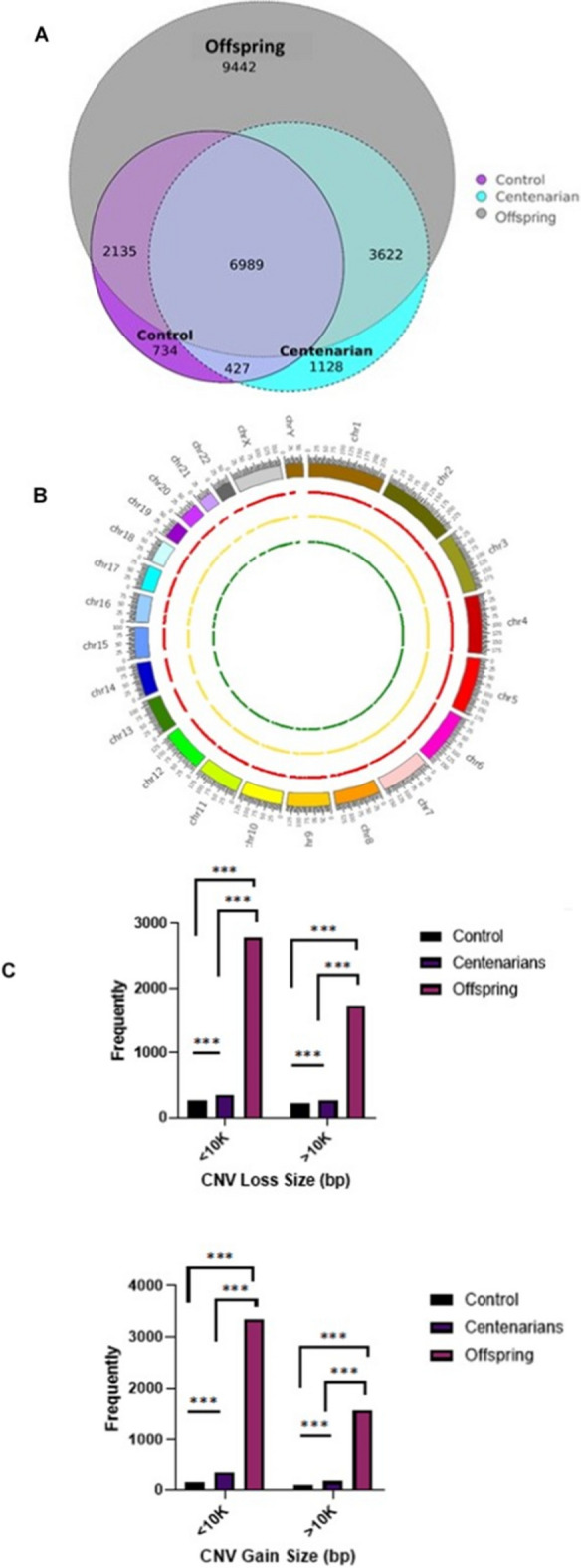
**A** Venn diagram depicting unique CNVs found in centenarians, offspring, and controls. Area outside the intersection of circles do not share any genuine overlap. **B** Chromosomal distribution of unique CNVs from the centenarians (red), offspring (yellow), and controls (green). **C** The frequency and size distribution of CNVs of different size groups among centenarians, offspring, and control, with gains and losses displayed separately

Experimental details on the discovery of CNVs using Affymetrix custom array targeting 44,639 CNVs are presented in Fig. 1. This analysis included three distinct groups: centenarians (*n* = 287, age 97.4 ± 2.8), their offspring (*n* = 153, age 66.5 ± 7.0), and unrelated controls (*n* = 230, age 69.2 ± 9.2). Of the targeted 44,639 CNVs, 12,166, 10,285, and 22,188 gain/loss CNVs were observed in the centenarian, control, and offspring groups, respectively. A subset of these CNVs, 11,038 in centenarians, 9551 in controls, and 12,746 in offspring, overlapped, resulted with unique CNVs 1128 in centenarians, 734 in controls, and 9442 in offspring (Fig. 2). The total number of both gains and losses of CNVs was significantly different between centenarians and both the offspring (*p* ≤ 0.0001) and control groups (*p* ≤ 0.0182). After removing common and overlapping variants, the average loss and gain of CNVs indicated that centenarians and their offspring had more CNV gains and losses (2.26 ± 5.06, 51.95 ± 112.4 respectively) compared to controls (1.1 ± 1.7). Intriguingly, both the offspring and centenarian group exhibited more than 26% among centenarians and almost eightfold among the offspring of more gain and loss in CNVs than the control. Similarly, CNV losses in the centenarian and the offspring groups were ~ 20% and 2.5-fold higher than those in the control. As a result, centenarians as a group displayed greater CNV gains and losses compared to unrelated control and fewer than their offspring. No statistically significant difference was found in CNV gains between the centenarian and the control groups (Table 1).

**Table 1:** Summary statistics of CNV of centenarians, progeny, and controls. Data was expressed mean ± SD; *NS* not significant, *Cn* centenarians,* O* offspring, *Ct* control

Characteristics	Centenarian (*N* = 287)	Offspring (*N* = 153)	Control (*N* = 230)	P for Cn vs. O	P for Cn vs. Ct	P for Ct vs. O
Age	97.4 ± 2.8	66.5 ± 7	69.2 ± 9.2	< 0.0001	< 0.0001	0.002
Gender	F-71.8%	F-55.5%	F-59.1%	0.0006	0.0026	NS
# of CNV gains	33.02 ± 104.16	430.63 ± 968.41	24.04 ± 54.29	< 0.0001	N.S	< 0.0001
# of CNV losses	53.14 ± 52.04	108.88 ± 206.1	44.47 ± 31.61	0.0002	0.0182	0.0014
# of CNV gain and losses	86.05 ± 120.06	539.51 ± 1074.71	68.41 ± 66.79	< 0.0001	0.0327	< 0.0001
Total lengths of CNV gains (bp)	98,643.1 ± 324,037.5	39,554.3 ± 328,430.6	86,405.8 ± 404,886.4	< 0.0001	< 0.001	< 0.0001
Total lengths of CNV losses (bp)	390,063.0 ± 2,933,009.7	139,508.5 ± 1,604,593.2	486,147.0 ± 3,520,988.1	< 0.0001	< 0.0001	< 0.0001
Total lengths of CNV gain and losses (bp)	296,354.1 ± 2,426,637.5	86,373.3 ± 1,125,093.4	379,374.1 ± 3,026,712.7	< 0.0001	< 0.0001	< 0.0001
Unique CNV gain and losses	2.26 ± 5.06	51.95 ± 112.4	1.1 ± 1.7	< 0.0001	0.0368	< 0.0001

We subsequently evaluated the total length of CNV gains and CNV losses separately, as the length provides a general offer and a general perspective on the extent of genomic material spanned by the CNV regions explored in our study (Table 1). The average total length of CNV gains in centenarians was approximately 0.1 MB, in contrast to 0.04 MB in their offspring, and 0.09 MB in the control group. Likewise, the average length of CNV losses was 0.49 MB in the control group and 0.14 MB in the offspring, compared to 0.39 MB for the centenarians. In terms of the length of genomic material, both the centenarian and offspring groups showed about 22% and 77% less CNV gains and losses compared to the control. The length of CNV loss and gains also differed significantly between centenarians and control for loss (*p* ≤ 0.0001) and gain (*p* ≤ 0.0001), respectively. These findings indicate that centenarians, despite being approximately three decades older, have gained or lost the least amount of genomic material—a metric either equivalent to or comparable with their younger counterparts. In short, centenarians appear to show relatively less genomic flux compared to their controls yet greater than their offspring.

### Unique CNV regions in each group

Centenarians, offspring, and controls showed 1128 (9.3%), 9442 (42.5%), and 734 (7.1%) mutually exclusive unique CNVs, respectively, as depicted in Fig. 2A (Supplemental Fig. 1). A concentric circular map of the genomic regions (Fig. 2B) of both gains and losses of unique CNVs among the three groups shows that the genomic loss/gain is distributed evenly across all chromosomes. The majority of the unique CNVs fell within a size range of < 10 kB. The incidence of CNV gain/loss was consistently highest in the offspring, followed by the centenarians and then the control group, for all CNV size classes considered (< 10 kB and > 10 kB).

**Fig. 3:**
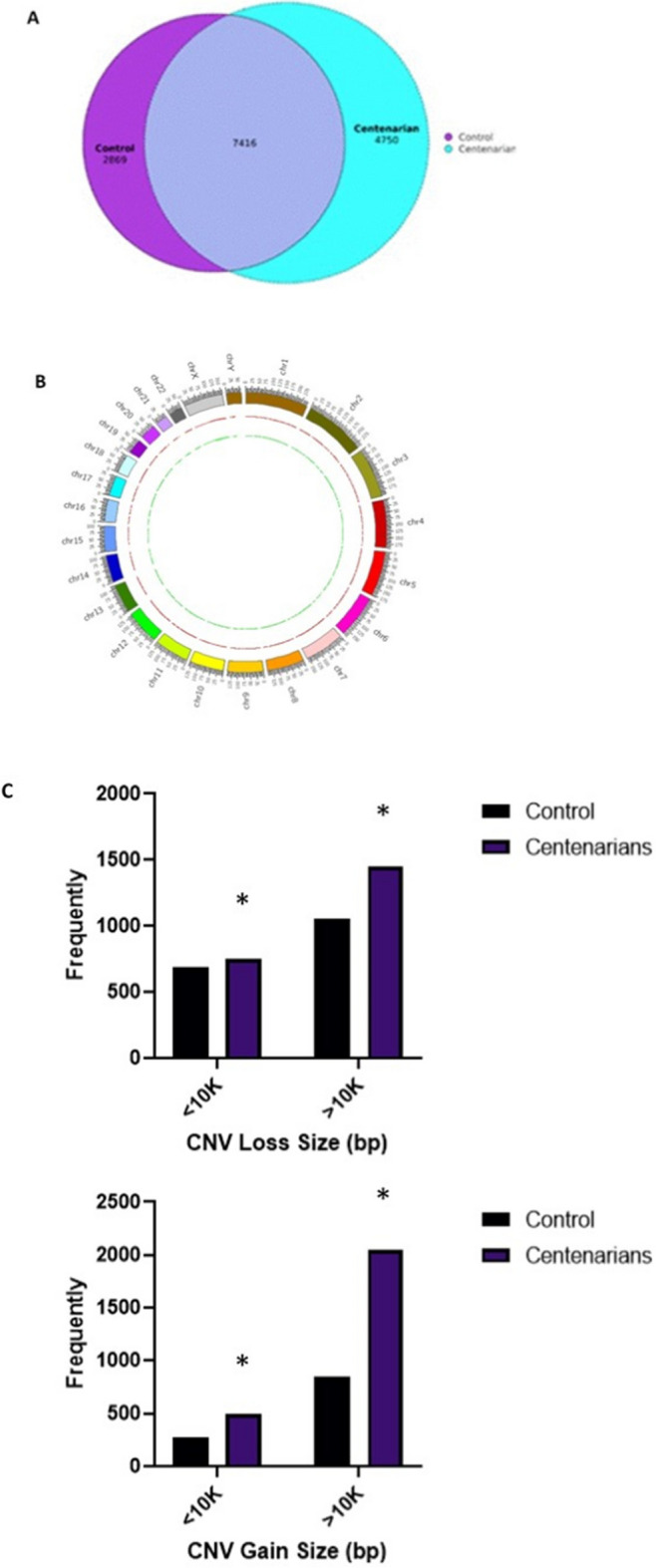
**A** Venn diagram illustrating unique CNVs found in centenarians and control. The inner light blue represents centenarians, and the pink represents controls. **B** Chromosomal distribution of unique CNVs from the centenarians (red), and controls (green). **C** The frequency and size distribution of CNVs of different size groups among centenarians, offspring, and control, with gains and losses displayed separately

Because the age distribution of offspring and controls used in this study are similar, we focused on the centenarian control comparison. Therefore, these overlapping CNVs were removed, and new chi-square analyses were performed, resulting in revised CNV counts for both groups. According to the updated data, while centenarians exhibited 4750 unique CNVs, the control group displayed only 2869 unique CNVs, as illustrated in the Venn diagram (Fig. 3A). The distribution of unique CNVs in terms of genomic gains and losses across chromosomes for both centenarians and controls is displayed in the circle graph (Fig. 2B).

Additionally, CNVs were categorized into two categories according to their length (< 10 kB, and > 10 kB). The majority of these unique CNVs fell within the size range of < 10 kB. Control individuals showed lower incidence of the gain of CNVs of all categories (< 10 kB, and > 10 kB), followed by centenarians. Notably, the difference in the size of gains and losses of large CNVs in centenarians was significantly distinct from that in the control group, as shown in Fig. 2 C.

Taking advantage of the presence in our cohort of centenarians and control, we identify one copy gain\loss and two copy gain\loss for each group. We examined the percentage of losses and gains of CNV in centenarians and controls. The lower percent obtained in gain plus 2 and loss minus 2 is 7 and 18%, respectively. Then there was an increase in loss minus 1 and gain plus 1, 19, and 29%, respectively. We then tested the frequency of CNVs between centenarians and controls. We divided all losses and gains for control and centenarians into subgroups (− 2, − 1, 1, and 2), and we observed that when the number of groups was large, the frequency of common CNVs was lower (Supplemental Fig. 2).

### Functional analysis of unique CNVs (between centenarians and the control group)

**Fig. 4:**
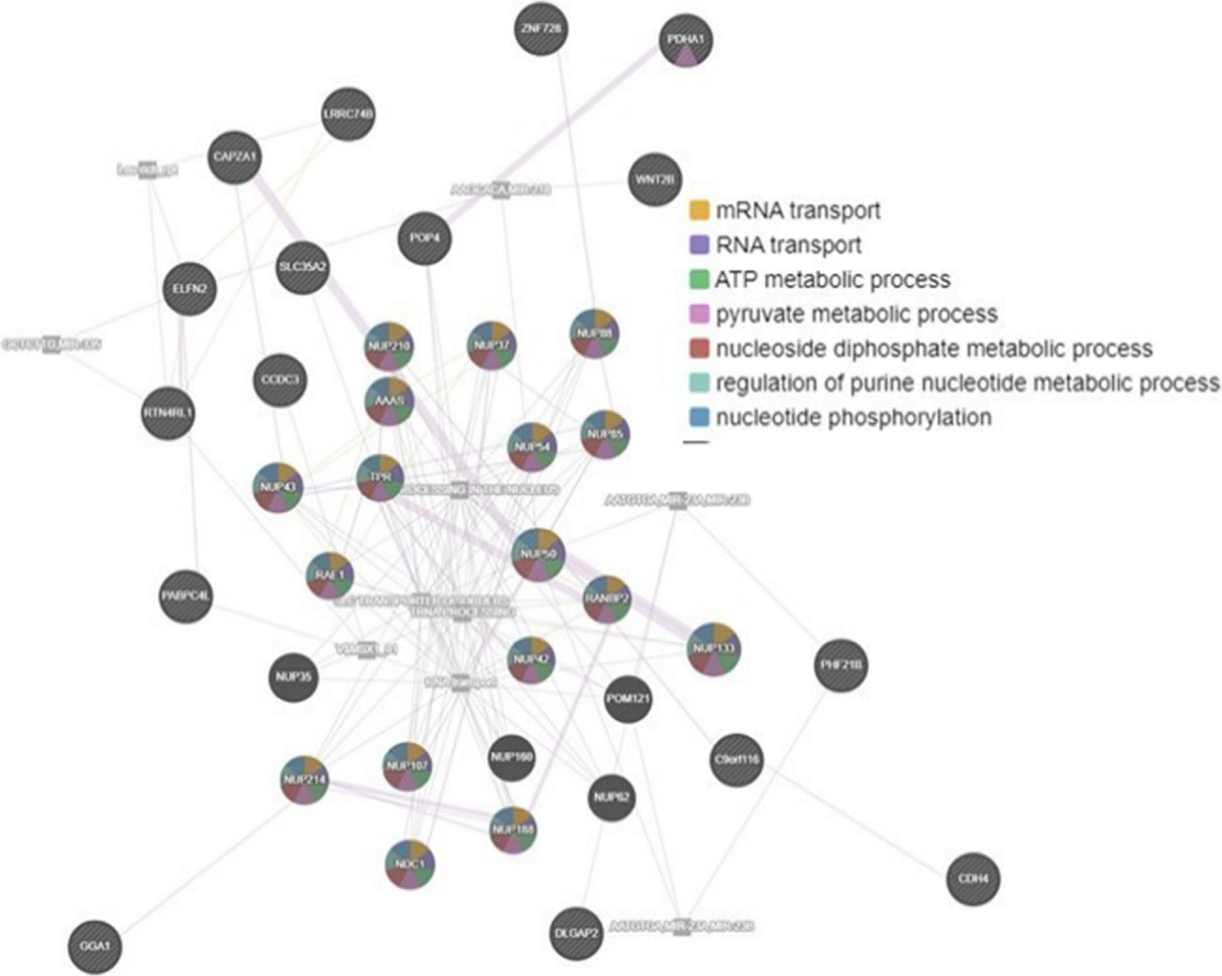
Functional assignment of genes in networks and pathways. GENEmania network showing co-expression patterns of genes, with each color indicating functional processes shared by genes

The assignment of genes into functional classes, as determined from our previous comparative analysis and computational approaches, is illustrated in Fig. 4. This classification is in concordance with the functions traditionally associated with each of the genes listed. Genes implicated in these metabolic pathways serve a broad range of cellular roles, including but not limited to physiology, proliferation, nucleic acid binding, DNA repair, immunity, transfer and transporter proteins, cell adhesion, and cell cycle regulation. Genes in these categories include *CAPZ1*, *MAPK1*, *SAA1*, and *NUP50*. Our stringent mapping revealed a significant enrichment, particularly within functional gene sets located in stable regions. One of the genes identified, MAPK1, is associated with aging-related processes, specifically in the context of neuroinflammation and Alzheimer’s disease (as shown in Fig. 3A). The top enriched pathways for this gene are depicted in Fig. 4, which include RNA transport, citrate cycle, vitamin B6 metabolism, glycolysis/gluconeogenesis, and RNA degradation.

### Analysis of the functional impact of CNVs on gene expression levels

**Fig. 5:**
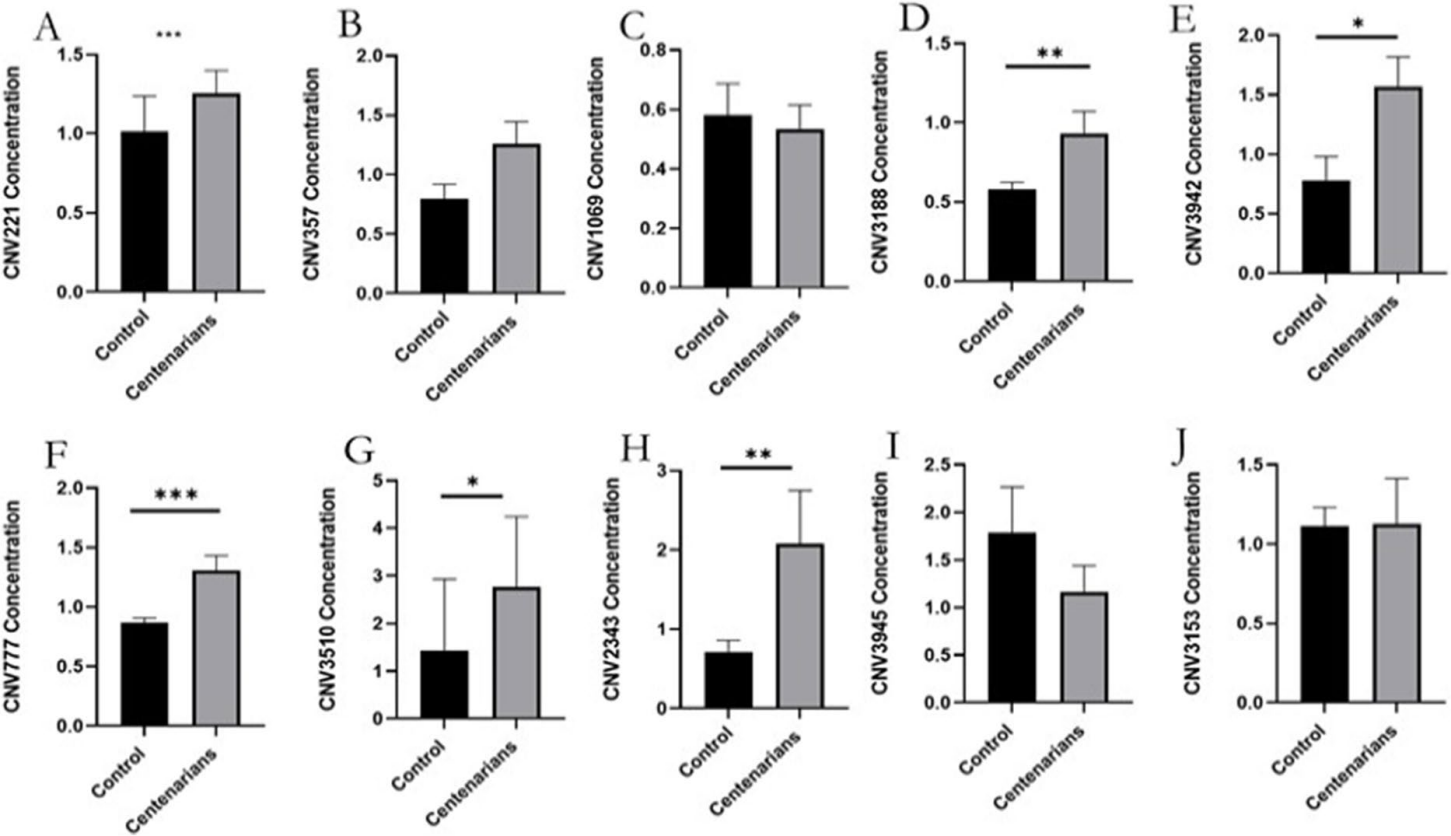
qPCR confirmation of differential expression levels of the CNV between centenarians and controls blood samples

The statistical and graphical analyses suggested that the distribution of CNVs vary across centenarian, progeny, and control groups. We sought to experimentally validate our statistical findings. We collected blood samples from both control subjects and centenarians. For ten specific CNVs (Table s3, Fig. 5), our data revealed a significant gain in copy number for six CNVs—CNV3188, CNV3942, CNV221, CNV777, CNV3510, and CNV2343—in long-lived individuals compared to the control group. We can show the enrichment result figure of genome content in a specific region (Figure Supplementary 2).

To determine if these six CNVs influence biological aspects of aging, we investigated their relationship with gene expression levels. Specifically, we aimed to discern any functional biases unique to CNVs that appear with significantly higher frequency in centenarians. Our analysis revealed several genes located within or adjacent to ten identified CNVs, including adaptor-related protein complex 2 subunit alpha 2 (*AP2A2*), pericentrin (*PCNT*), dynein heavy chain 17, Axonemal (*DNAH17*), phospholipase A and acyltransferase 1 (*PLAAT1*), cystatin-like protein (*CYS*), lanosterol synthase (*LSS*), and tumor necrosis factor ligand superfamily member 4 (*TNFSF4*).

**Fig. 6:**
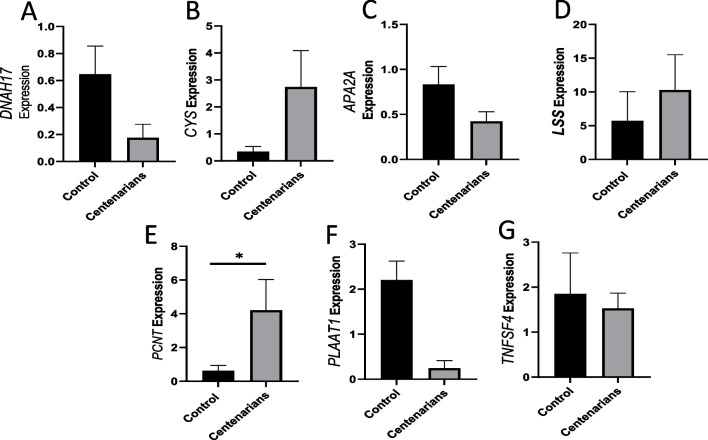
Relative quantitative expression of genes between control and centenarians

Some of these genes have previously been linked to aging and the involvement of genes in a wide range of biological processes involved in aging. Age-stratified analyses showed that *LSS* gene was positively associated with aging [26], and lipid metabolism. It also appears to play a pivotal role in human longevity and healthy aging [23]. To further investigate the differential expression of these genes, we employed RT-PCR to compare gene expression levels between centenarians and controls. Our results revealed an upregulation of the *PCNT* gene, while the expression levels of the other candidate genes did not show a statistically significant change (Fig. 6).

## Discussion

Extreme longevity and superior health in centenarians have been the subject of numerous investigations. Both genetic and environmental factors have been suggested to influence exceptional lifespan in humans [2, 16, 27]. Our research, focusing on CNV within an Ashkenazi centenarian cohort, offers novel insights into genome variation as it relates to aging.

The objective of this study was to discover patterns of genomic variants among three distinct groups, i.e., centenarians, their offspring, and a control group, using a well-structured, three-way design. We employed a custom Affymetrix array for this purpose which enabled high-resolution detection of CNVs down to 1.0 kB. This approach contrasts with a previous study on CNV and aging, which was limited to larger variants [14]. While these investigators reported an association between the burden of large deletions (> 500 kB) and higher mortality [14], our investigation also explores the potential influence of small (< 10 kB) and medium-sized (>10 kB) CNVs on longevity, comparing centenarians with their offspring and controls. The centenarians included in our study were at least 20 years older than their offspring and the control group. The study revealed that centenarians exhibit distinct patterns of gain and loss in CNVs when compared to their descendants and control groups. We suggest that these unique patterns of genomic material may contribute to the differing longevity observed between centenarians and younger individuals. Notably, our data show that centenarians generally have lower length of both CNV gains and losses compared to control, implying that at least certain regions of their genome have remained relatively stable throughout their long lives as depicted from their offspring. This finding suggests that prolonged genomic integrity (greater resistance to genome flux) could be a key factor in their extended lifespan. Our results are in close alignment with Erceg et al.’s study on chromosomal structural variation [16]. These authors proposed that a low level of genomic variation likely contributes to genetic stability of these individuals and may also contribute to survival into advanced ages. Stoeger et al. similarly reported a positive correlation between transcript length and longevity [18]. It is reasonable to assume that very old people had a slower rate of accumulation of genetic damage throughout their lives as proposed recently [23]. Thus, the relatively low level of chromosomal aberrations in the “oldest old” people is likely to be both a consequence of their genetic stability and a contributing factor to their attainment of advanced age. One advantage of family-based study designs is their capability to differentiate between de novo and inherited CNVs, once an association between CNV and phenotype is shown. Certain studies have shown that there is an increased prevalence of pathogenic CNVs that are inherited from the mother, but the level of maternal transmission is not universal. The study of Leppa et al. found that in multiplex families, there is a decrease in the rate of large, rare de novo CNVs, and an increase in the number of large, rare inherited CNVs [28–32]. There is another aspect of counting CNVs; while looking at the pattern in offspring compared with their parents (Supplementary Fig. 2 Supplementary Table 4), we see that there are almost 10% (1613) inherited gain–loss CNVs but almost ten times more gain–loss CNVs that are unique (16,936). Since we hypothesize that the offspring mimic their parents’ CNV counts when they were at the same age, there is CNV dynamic of lost and gain during the years. Thus, those that are present today among the offspring might be lost or gain among the centenarian along the year. According to the overall analysis of the 153 offspring, the centenarian father and son are found in autosomal chromosomes with CNVs, including all the gains and losses of CNVs. Such dynamic can be seen in table S4 in which almost 50% have lost the parents’ trend. In summary, the genomic features of centenarians largely resemble those found in the general middle-aged population, designating them as a unique subset of the elderly whom we might call the “privileged ones.” Our findings further extend to model organisms; for instance, Erceg et al. reported that wild-type worms, which have a normal lifespan, accumulate larger numbers of copy variations in their genomes compared to the long-lived daf-2 strain [16]. These findings are consistent with a lower frequency of short CNV losses observed among the younger cohort, underscoring the notion of greater genome stability in centenarians. Furthermore, the moderate levels of CNV gains and losses observed in the descendants highlight the genetic influence of their centenarian parents, an impact that becomes less discernible when focusing solely on unique segments. Our data indicate that approximately 25% of the CNVs in our cohort were individually uncommon; half of these were unique occurrences, and the rest were observed no more than twice.

We also found that controls showed excess CNV gain and loss in regions containing genes such as *GPSM1*, *SLC35A2*, *PDHA1*, *NUP50*, *CAPZA1*, *RTN4RL1*, *FBXO25*, *PDXP*, *RNF130*, *KLRK1*, *ERICH1*, and *GGA1*, relative to centenarians. These genes are involved in RNA transport, DNA repair, cellular developmental, and transcription factors. Briefly, *SLC35A2* is a known transporter of UDP-galactose to the Golgi apparatus, and necessary for the glycosylation [33]. *CAPZA1* promotes HCC cells by regulating the remodeling of F-actin. Furthermore, *CAPZA1* is an actin-binding that can bind to the barbed ends of actin filaments, maintaining the stability of the actin cytoskeleton [34]. *PDXP* plays a key role in regulating of vitamin B6 levels [29]. Further, *GGA1* is critical to liver-stage parasite development [35]. KEGG pathway analysis demonstrated that the upregulated differential expression genes were enriched in significant pathways (Fig. 4), including transporter and metabolism, whereas the downregulated differential expression genes were enriched in different pathways (Fig. 4), including glycolysis/gluconeogenesis and degradation. Among all GO terms, the most significantly upregulated differentially expressed genes were *POP4* and *NUP40*, and the most significantly downregulated differentially expressed genes were *PDHA1* and *PABPC4L*.

Additional lines of evidence further substantiate the unique inheritance patterns observed among centenarians. Consequently, it is plausible to hypothesize that male offspring born to centenarian parents may have an increased likelihood of reaching centenarian status themselves. This contrasts with offspring resulting from unions between an individual born to centenarian parents and another individual born to parents with a normal lifespan (Supplementary Fig. 1 and Supplementary Table 1).

Single-nucleotide polymorphisms (SNPs) correlate positively with exposure to common diseases through genome-wide association studies (GWAS). However, the genetic component of most common diseases is not fully explained by the SNP associations that are currently available, leading to significant conjecture regarding the missing heritability.

Craddock et al. assumed a genome-wide association study of common CNVs in eight diseases by developing a novel array targeting the highest-discovered set of CNVs. Their findings update our understanding of the genetic influences on common diseases, offer methodological insights into CNV analysis, and require a resource for human genetics research. They identified numerous CNV loci that are linked with common diseases. Such loci could contribute to disease pathogenesis. Nonetheless, the loci identified are fully tagged by SNPs, and thus, the associations can be ultimately detected via SNP association studies [36]. CNVs encompass more total bases and seem to have a higher mutation rate and possibly greater effects on gene structure, gene regulation, and consequently gene expression compared to SNPs.

While our study offers valuable insights into genomic plasticity as it relates to longevity, it has some limitations. First, our comparative analysis of genomic variants among centenarians, their offspring, and control subjects suggesting that the relatively small sample size may influence the accuracy of data prediction. Small sample sizes can pose potential challenges in terms of overfitting and generalizability to larger sample sets. We chose to exclude related individuals due to issues associated with identity by descent (IBD). However, incorporating siblings of centenarians and larger family clusters could yield more comprehensive data on the pattern of inheritance and distribution of genomic variants that influence longevity within families of centenarians, especially the influence of IBD fragments. From this perspective, it is worth noting that siblings of centenarians have been shown to have longer lifespans than the general population [37]. We also identified genomic regions ranging in size from approximately 2 to over 100 kB that do not contain any known genes, leaving their roles in the context of longevity undefined. Nevertheless, recent findings from the ENCODE (Encyclopedia of DNA Elements) project suggest that intronic and intergenic regions may indeed have functional significance, potentially influencing development.

Computational analyses provided important insights on the causal and coordinated relationship among genes involved in cell physiology and function in relation to aging in this cohort; these need to be confirmed using functional genomic approaches across critical stages in the human lifespan across diverse ethnic groups. RT-PCR is the most frequent method used for this purpose. After all, this method requires optimization of PCR conditions and even when this is done, results are not always honest; in contrast, the methods we define here are highly accurate and reproducible. While several methods are available to detect CNVs in genomic sequences, all may be expected to give false positive results. As any change in the quality of the DNA samples of detection methodology is likely to give a result consistent with loss or gain of copy number. In this study, we identified 10 CNVs associated with long-lived Ashkenazi centenarians including eight duplications and two deletions. These CNVs might affect the expressions of more than 10 known genes; some of these genes were known to regulate the cellular aging process.

CNVs serve as a significant source of variations in the human genome, contributing both to population diversity and to the genetic basis of human diseases [38]. In our study, we identified variations in the gain/loss of several genes—*CYS*, *PLAAT1*, *LSS*, *DNAH17*, *TNFSF4*, *APA2A*, and *PCNT*—to examine disease predisposition from a CNV standpoint. Among these, the *LSS* gene has been identified as playing a critical role in human longevity and healthy aging [39]. Given its involvement in a variety of fundamental biological processes, this gene may also have a broader influence on aging-related phenotypes. While human *CYS1* is a promising candidate for the development of cystic kidney disease [40], earlier studies showed that increased total *CYS* is associated with pathologic conditions such as cardiovascular diseases [38], which in the mitochondria is an essential cofactor for proteins in mitochondrial respiration and other cellular activities [39]. Gene alterations of *PCNT* are associated with a type of primordial dwarfism, intrauterine growth retardation, cardiomyopathy, and early-onset type 2 diabetes [41]. Malfunction of *PCNT* gene causes premature aging, brain development, and inflammatory- and immune-related Down syndrome–related responses associated with *PCNT* mutations [42]. Overexpression of *DNAH17* by downregulation of methylation levels might contribute to hepatocellular carcinoma (HCC) initiation and progression [40]. In addition, the hypomethylation status of the *DNAH17* gene, both in tumor tissue and in adjacent non-cancerous tissue, could be a promising biomarker for tumor thrombosis in HCC. Further, it was found that *DNAH17* methylation was associated with gender, age, serum AFP values, liver cirrhosis, tumor fibrous capsule, tumor necrosis, and tumor thrombus [43]. Another study revealed a novel missense mutation of *DNAH17* resulting in morphological abnormalities of the sperm flagella (MMAF) phenotype. *DNAH17* is evolutionarily conserved in many species and is dominantly expressed in the testis. Earlier studies have indicated that *DNAH17* plays a significant role in sperm motility in humans and mice [44]. More importantly, the *DNAH17* gene decreased with increasing age.

Previous research has demonstrated that polymorphisms in the *TNFSF4* gene can increase susceptibility to various autoimmune diseases. Moreover, *TNFSF4* has been implicated in the development of atherosclerosis [39]. Interestingly, one study found that the expression levels of *TNFSF4* in a general population did not correlate with age [41]. Atherosclerosis, in general, contributes to reduced life expectancy. The AP2A2 gene, which has been associated with Alzheimer’s disease, is widely transcribed in human tissues, particularly the brain. It plays a central role in regulating clathrin-mediated endocytosis and has also been identified as influencing risk for the development of cardiovascular diseases [42].

To summarize, our study suggests that genome plasticity and integrity are interconnected aspects of the aging process [6, 44]. Beyond the well-studied genomic regions such as telomeres [22], sub-telomeric regions [9, 45], and duplicated genes like *FOXO* and *SIRT* [24, 46, 47], a number of other genes embedded in genomic regions also play a major role in influencing senescence and longevity. These regions exert a direct, indirect, and cumulative impact on both the health and longevity of individuals. A comprehensive analysis comparing the genomic regions, their inheritance patterns, and the functional role of genomic regions between centenarians and controls could offer valuable insights into the human aging process. Given the socio-economic significance of aging populations, a nuanced understanding of the genomic factors affecting key life stages could pave the way for identifying age-specific biomarkers [37] and targeted multi-stage interventions aimed at improving health span [1

### Experimental procedures

#### Study population and design

The study utilized the Ashkenazi Jewish cohort (AC), which was derived from the larger Ashkenazi Jewish population. Specific details on the demographic genetic history and the appropriateness of the Ashkenazi Jewish population for genetic studies are discussed in other publications [22, 23]. The study participants were recruited by advertising in Jewish aging centers. In accordance with the policy of the Clinical Investigation Committee of the Albert Einstein College of Medicine, informed consent was obtained. A physical examination and a medical history report were conducted by a therapist who visited all the participants. This included a review of the questionnaire. If there were any discrepancies in the family history data that could not be resolved by agreement in the presence of the known and offspring who completed the questionnaire, then the data was not used. The AC is comprised of three distinct groups: centenarians, their offspring, and unrelated controls. One advantage of this tripartite design is its ability to minimize the issue of “longevity contamination” in the control group. By selecting controls whose parents did not reach exceptionally long lifespans (i.e., ≤ 85 years), we reduce the risk of conflating the “younger samples” with the centenarians. Comparing centenarians and their offspring helps in identifying at least some shared copy number variants (CNVs) between these two groups. As the age distribution of the offspring and the control groups is similar, the offspring serve as both an internal check and a proxy for the centenarians, providing a consistent basis for comparison with the control group. For our analysis, we employed Affymetrix technology (Affymetrix Genome-Wide Human SNP Array *6.0*), using genomic DNA extracted from 143 individuals who represent each of the three groups. According to unpublished data by Ionita-lonza et al., a sample size of approximately 40 individuals is sufficient to discover around 95% of CNVs with a population frequency greater than 5% [48]. To visualize unique CNVs among the groups, we used the eulerr program to generate a Venn diagram.

### Checking for IBD

The demographic history of the Ashkenazi population suggests a tendency for close biological relationships, resulting in the sharing of genomic segments IBD among some individuals. Given that a high degree of relatedness can compromise the integrity of association studies [49], it was essential to exclude individuals with high levels of IBD segment sharing from our analysis. To this end, we employed the GERMLINE algorithm [50] to remove closely related individuals from both the centenarian and the control groups. The algorithm identifies two individuals as relatives if their total shared genomic material exceeds 1500 cM and the average length of shared segments is greater than 25 cM. It is worth noting that the offspring of centenarians serve as immediate descendants and are used solely as a proxy for the centenarian group in our study. Because the offspring of centenarians, by definition, are the immediate descendants of centenarians, we used them only as a proxy for centenarians; each of these individuals are unrelated by the IBD criteria (considering that socioeconomic and genetic background is similar for progeny from the same family).

### DNA source, extraction, and processing

The DNA samples used for the Affymetrix study were obtained from intravenous blood samples, according to the guidelines approved by the Institutional Review Board of the Albert Einstein College of Medicine (AECOM) of Yeshiva University. A total of 10 ml of blood was collected in 2–3 acid citrate dextrose tubes from each individual enrolled in the study and stored in the Human Genetics Core Facility at AECOM according to the established protocols. DNA was isolated from the stored blood using the Puregene DNA Purification System (Gentra system, Minnesota) according to the manufacturer’s instructions and stored in aliquots at − 40°C. qPCR analysis was performed on genomic DNA in differentially CNV interval identified as a result of the comparison between Ellis centenarians and controls. Statistical significance between groups is determined by the Wilcoxon test (*p < 0.05, *p < 0.01). We used *RNASE-P* as a housekeeping gene to normalize the measurement.

### RNA extraction

Total RNA of the blood samples were extracted using GeneJet RNA Purification Kit (Thermo Fisher Scientific USA, in order to determine gene expression levels, using RNeasy Mini kit (Germany) and High Pure PCR Template Preparation Kit (Roche, Mannheim, Germany). Isolated RNA was quantified using Nanodrop-1000 spectrophotometer (Nanophometer® Np80, IMPLEN) and 1 μg RNA was reverse transcribed into cDNA using High-Capacity cDNA Reverse transcription Kit (Applied Biosystems, CA, USA), using a random primer scheme for initiating cDNA synthesis. We experimentally validated some significant CNV using (qPCR), which is a commonly used technique to establish functional relationships of genes. For transcript abundance analysis of candidate gene: PCNT, AP2A2, DNAH17, TNFSFS4, PLAAT1, CYS, and LSS using SYBR Green master mix (Applied Biosystems, USA). PCR reactions were performed under the following conditions: 10 min at 95 °C and 40 cycles of the one-step thermal cycling of 3 s at 95 °C and 30 s at 60 °C in a 96-well reaction plate. Specificity of PCR reactions was verified by melting curve analysis of each sample amplified product. Results were normalized to the expression of housekeeping gene *HPRT* for RNA samples and *RNASE-P* for DNA samples*.* To identify the CNVs associated with longevity in Ashkenazi centenarians, we recruited ten long-lived individuals and ten middle-aged individuals to serve as controls. The primers used were designed using Primer3 + web tool (http://bioinfo.ut.ee/primer3-0.4.0/) and are listed in Supplemental Tables S1, 2.

### Genotyping console

We used the Birdseed software to generate cell files for samples that passed the initial Affymetrix 6.0 array QCs. Soon after, the Genotyping Console (GTC v.3.0.2) was implemented to allocate CNVs with default parameters of 1-kB size and five probes.

### Discovery of candidate CNVs using Affymetrix array

We used Affymetrix Genome-Wide Human SNP Array 6.0 microarray targeting 44,639 distinct CNV regions, in the study, and followed the manufacturer’s instructions for the discovery and visualization of the genomic variants. Briefly, the experimental procedure is as follows. Genomic DNA (1500 ng) samples used in the test and reference (A10851) received from the Einstein College of medicine, NYC, NY, were fragmented using heat fragmentation. Genomic DNA samples were genotyped using Affymetrix Genome-Wide Human SNP Array 6.0 platform (Affymetrix), according to the manufacturer’s protocol. The genotype calls of each individual were determined by the Birdseed version 1 genotype calling algorithm, embedded in the software Affymetrix Genotyping Console 2.0 (Affymetrix) which was implemented to allocate CNVs with default parameters of 1-kB size and five probes. Top significant CNV regions that passed the FDR were validated using quantitative PCR (qPCR). It is critical that gains and loss of CNV regions discovered by computational approaches are confirmed using molecular methods. Accordingly, primer assays for regions that passed the FDR were designed using primer 3.4 web interphase program (http://frodo.wi.mit.edu/primer3/). Thirty-five CNVs were randomly selected and a total of *143* samples and an *additional 650 Korean samples* were used to evaluate the sensitivity of custom Affymetrix and it was 0.87 [38].

Briefly, the qPCR mixture consisted of 25 ng of genomic DNA, 2 × of SYBR, 50 × of ROXII reference dye, and 10 μM of primers in a 20 μl total reaction volume. Each experiment was run in triplicate. PCR reactions were incubated for 2 min at 95°t followed by 40 cycles of 5 s at 95°f and 30 s at 60°a. Data were processed with the SDS 2.3 software using the standard DDCt method. The qPCR and Affymetrix results were compared for consistency. In each validation region, samples were clustered into three groups (CN loss, normal, and gain) by DDCt values and the corresponding log_2_ ratios independently and a 3 × 3 table was generated. We also validated some of these regions by regular PCR with flanking primers targeted against the ends of each CNV region. PCR amplification was performed in 50 μl with 50 ng of genomic DNA, 10 pmol of forward and reverse primer each, standard volume of Ex Taq (Takara), Ex Taq buffer (Takara), and dNTPs (Takara) at 95° (for 10 min, 40 cycles of 95° for 30 s, 60° for 30 s, 72° for 30 s, and finally, 72° for 10 min).

## Statistical analysis of CNV data

We employed Fisher’s exact test to find differences in the distribution of gain or loss of specific CNV regions among the two groups: centenarian and control; centenarian and progeny; control and progeny. Each CNV is examined by using the asymptotic chi-square test. All statistical comparisons were made using SAS.9.1.

## Network and pathway analyses

We used the web server of the GeneMania Consortium, http://genemania.org, to perform an analysis that assigned functions to the genes that showed significant departures among centenarians and controls. Gene-set enrichment results were visualized as a network in order to group highly overlapping gene sets into functional clusters.

All results for CNV and gene expression are displayed as means ± S.E. *t*-test was used for the evaluation of the differences for all parametric data or its non-parametric equivalent, Wilcoxon test. *p* < 0.05 was considered significant. JMP 15 and GraphPad Prism 8 software were used for the calculation and drawing of the results.

### Supplementary Information

Below is the link to the electronic supplementary material.

Supplementary file1 (DOCX 605 KB)

Supplementary file2 (XLS 38 KB)

Supplementary file3 (DOC 33 KB)

Supplementary file4 (XLSX 610 KB)
