# Correction to: Genome integrity as a potential index of longevity in Ashkenazi Centenarian’s families

**DOI:** 10.1007/s11357-024-01253-6

**Published:** 2024-06-18

**Authors:** Mariana Andrawus, Gil Ben David, Ivana Terziyska, Lital Sharvit, Aviv Bergman, Nir Barzilai, Srilakshmi M. Raj, Diddahally R. Govindaraju, Gil Atzmon

**Affiliations:** 1 Faculty of Natural Sciences, University of Haifa, Haifa, Israel; 2 Sagol Department of Neurobiology, Faculty of Natural Sciences, University of Haifa, 199 Aba Khoushy Ave., 3498838 Mount Carmel, Haifa, Israel; 3 Cornell University, Ithaca, NY 14853 USA; 4 Department of Systems and Computational Biology, Albert Einstein College of Medicine, Bronx, NY 10461 USA; 5 Departments of Medicine and Genetics, Albert Einstein College of Medicine, Bronx, NY 10461 USA; 6 Department of Genetics, Albert Einstein College of Medicine, Bronx, NY 10461 USA; 7 MCZ, Harvard University, Cambridge, MA 02138 USA

**Correction to: GeroScience**

10.1007/s11357-024-01178-0

The original version of this article unfortunately contained an error in the main body text.

The following text should be omitted in the last sentence of the first paragraph under the following title at the Experimental procedures section under sub-heading “Discovery of candidate CNVs using Affymetrix array”.

Thirty-five CNVs were randomly selected and a total of 143 samples and an additional 650 Korean samples were used to evaluate the sensitivity of custom Affymetrix and it was 0.87 [38].

