# Supplementary material for: Genome integrity as a potential index of longevity in Ashkenazi Centenarian’s families
Source: GeroScience. 2024 May 9;46(5):4147–62. doi: 10.1007/s11357-024-01178-0 (PMC11335978; doi:10.1007/s11357-024-01178-0)

Supplementary 4:

Additional lines of evidence further substantiate the unique inheritance patterns observed among centenarians. In parent-son relationships involving a centenarian father and a son from a spouse with a typical lifespan, the genomic inheritance pattern suggests the possibility of segregation distortion, particularly when *de novo* mutations are absent. Traits related to viability and fitness, such as longevity, are influenced by differential transmission of chromosomal regions or so-called "segregation distortion" [36]. Interestingly, there are relatively limited inherited CNVs per genome (approximately 160), and de novo germline CNVs are a well-known cause of critical congenital malformations and neurodevelopmental conditions. The rate of CNV mutations varies by several orders of magnitude depending on the genomic region and parent of origin because of differences in the mechanism by which the CNV is formed de novo. Consequently, it is plausible to hypothesize that male offspring born to centenarian parents may have an increased likelihood of reaching centenarian status themselves. This contrasts with offspring resulting from unions between an individual born to centenarian parents and another individual born to parents with a normal lifespan. As can be seen from table 4 the overlapped CNV with direct transmission is almost equal (parents son/daughter relationship) around 53%, the only pair that is significantly different is Mother Centenarian and son which is 59% suggesting better transmission and less variability within this paired inheritance.

Supplementary table 4: Counting CNVs pattern in offspring compared with their parents.

| CNV | | ELLI Female | | ELLI Male | |
| --- | --- | --- | --- | --- | --- |
|  |  | Daughter | Son | Daughter | Son |
| Gain | Gain | 175 | 240 | 273 | 136 |
| Gain | Loss | 141 | 160 | 244 | 110 |
| Loss | Gain | 531 | 306 | 302 | 293 |
| Loss | Loss | 563 | 441 | 376 | 331 |
| Total | | 1410 | 1147 | 1195 | 870 |


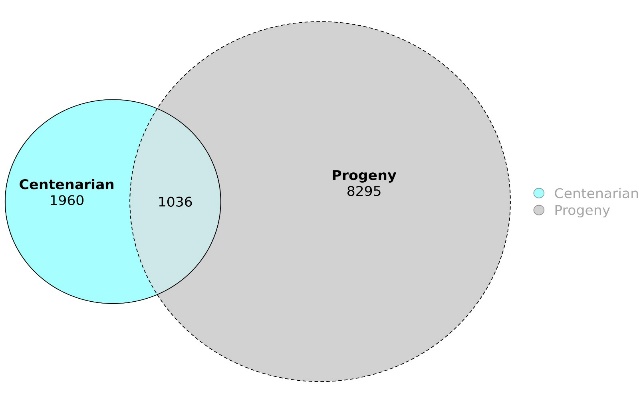

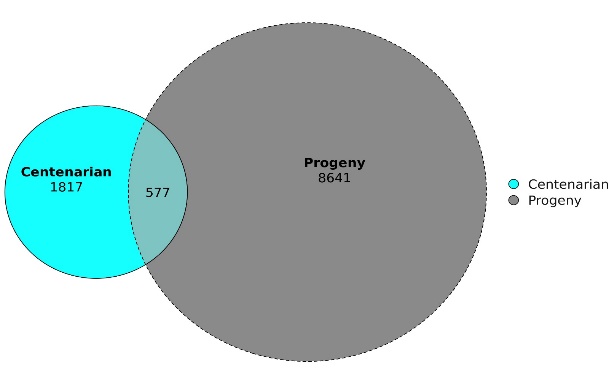

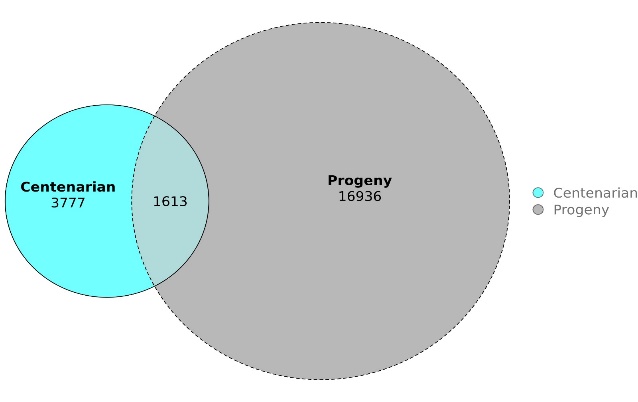


Supplementary Figure 1: Inherited loss-gain CNV vs. unique gain-loss CNV pattern in offspring compared with their parents.

Figure supplementary 2: Visualization of the enrichment of genome contents in the CNV region VarSome Stable-API v.9.4.6.

VarSome genome browser. A) Sequence (zooming in shows individual base pairs) and position. B) Transcripts’ exonic structure and orientation. C) Regions of interest in the protein (binding sites, functional domains, etc.) obtained from UniProt. D) Lollipop graphs indicating the pathogenicity of known variants in the region.

CNV3153


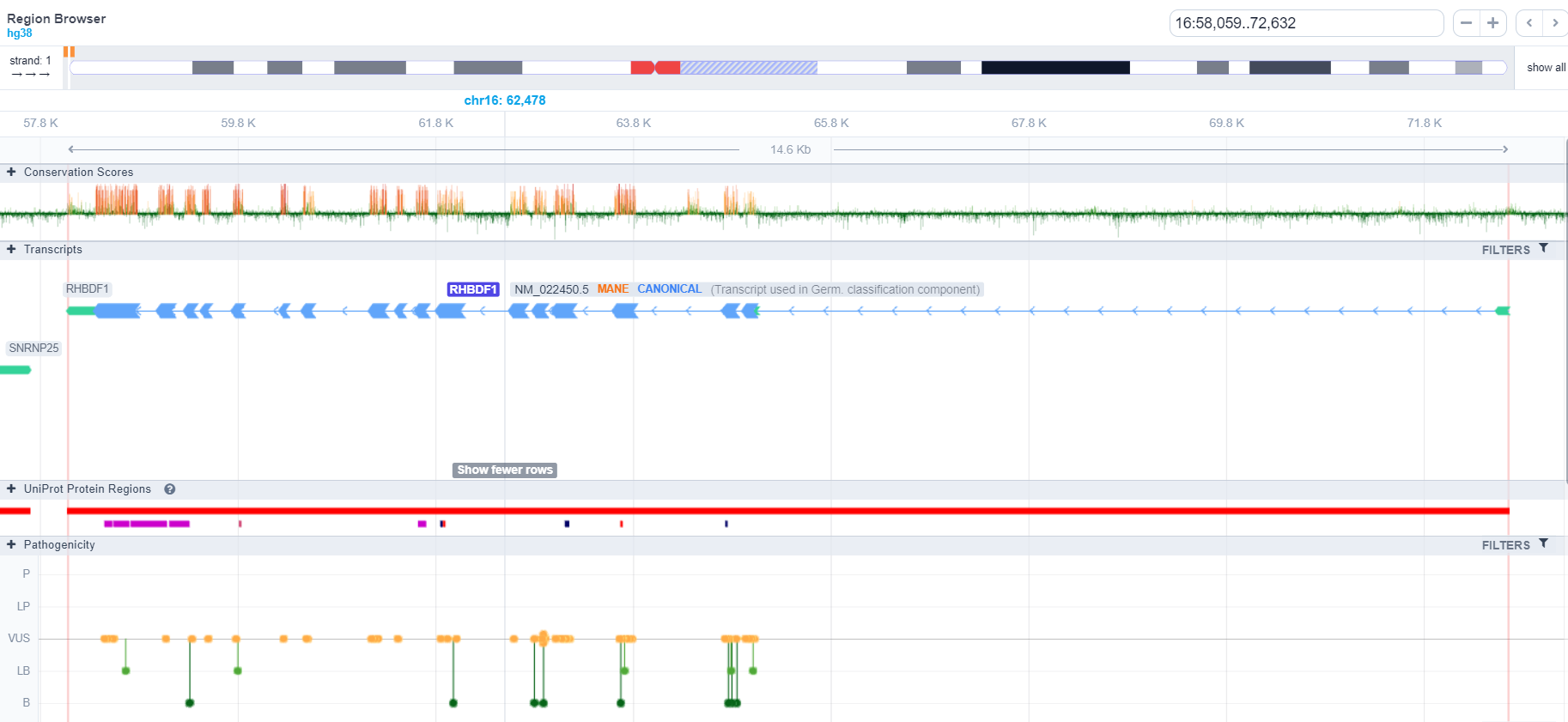


CNV357


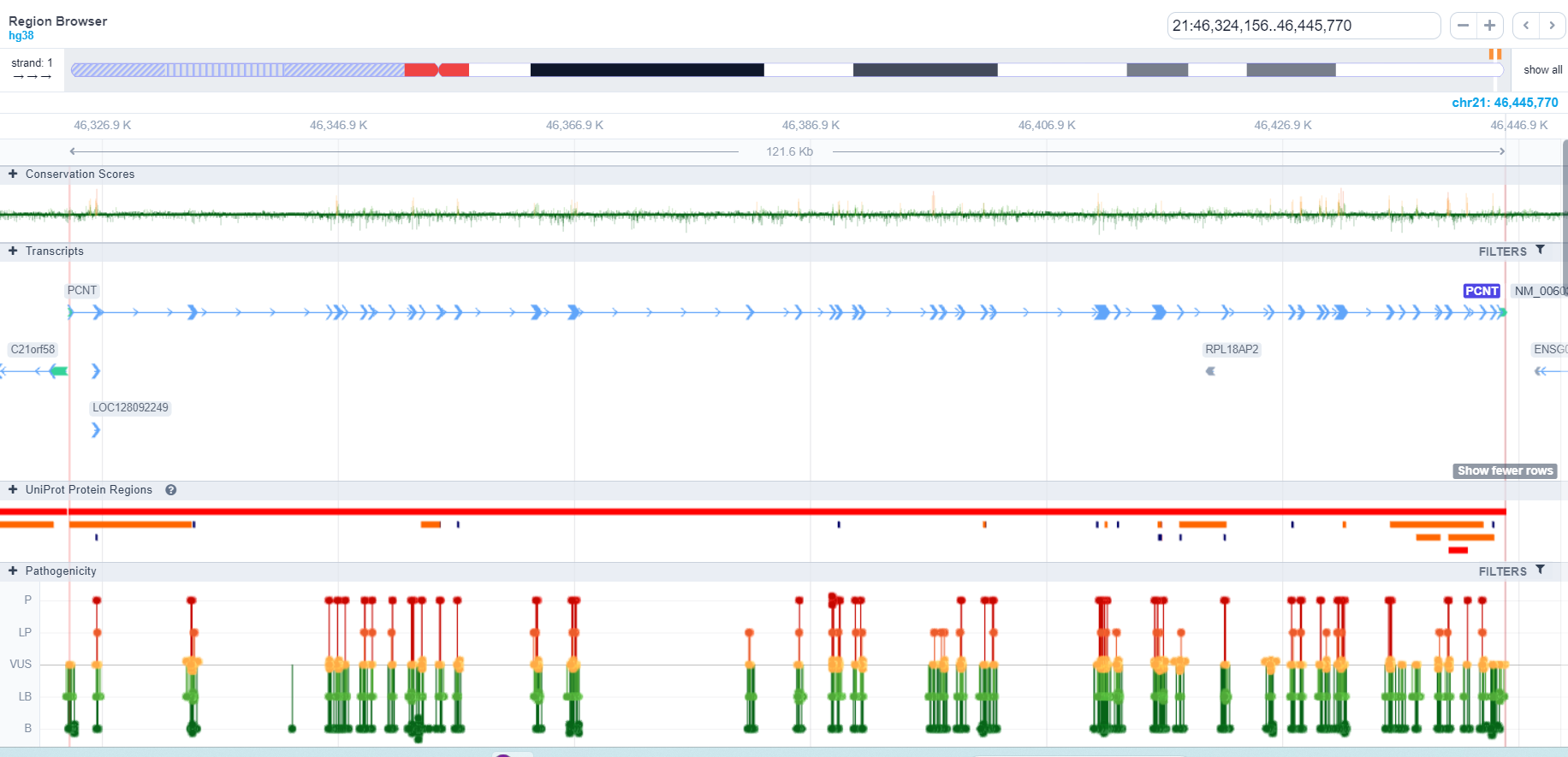


CNV3945


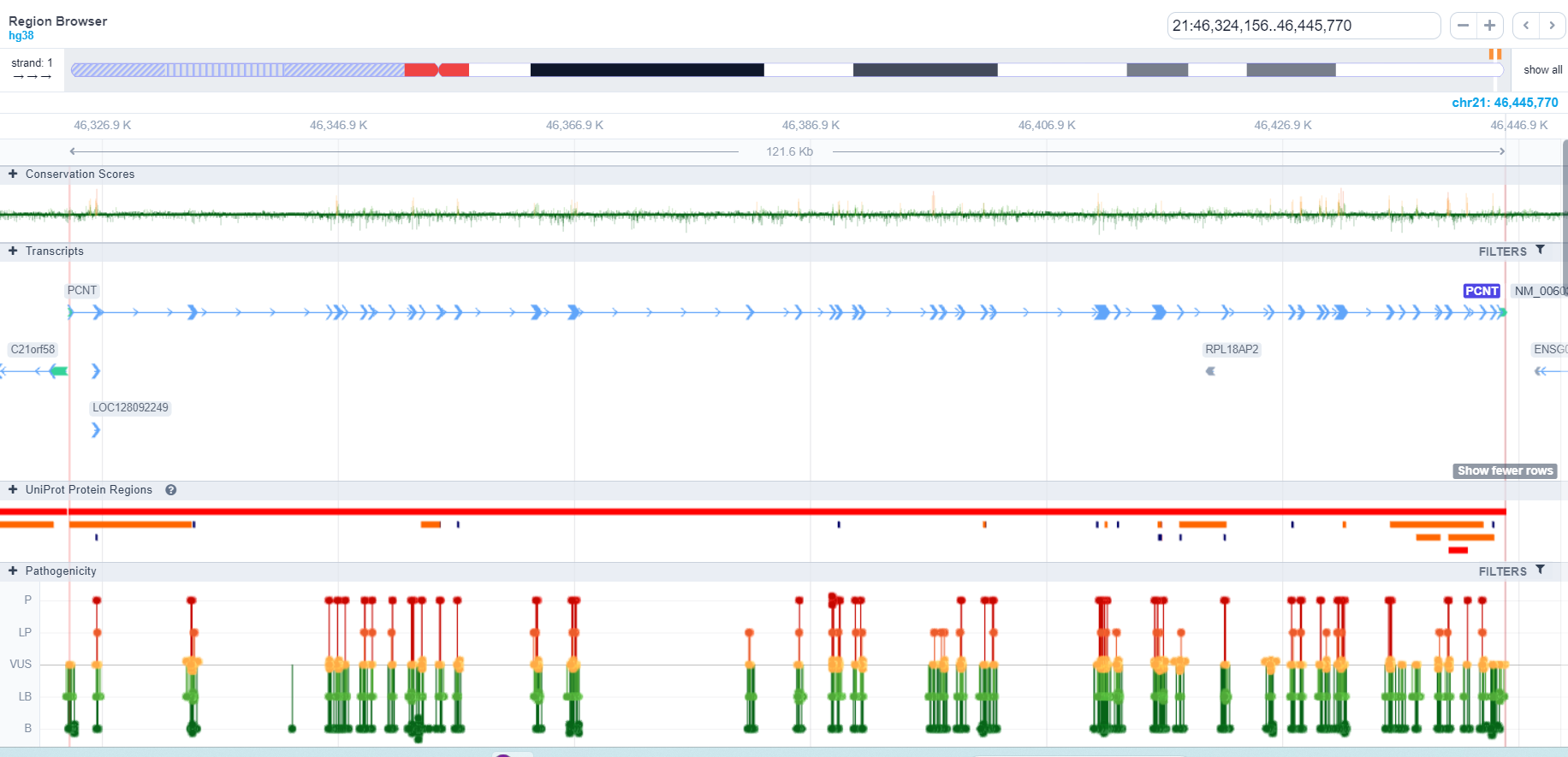


CNV2343


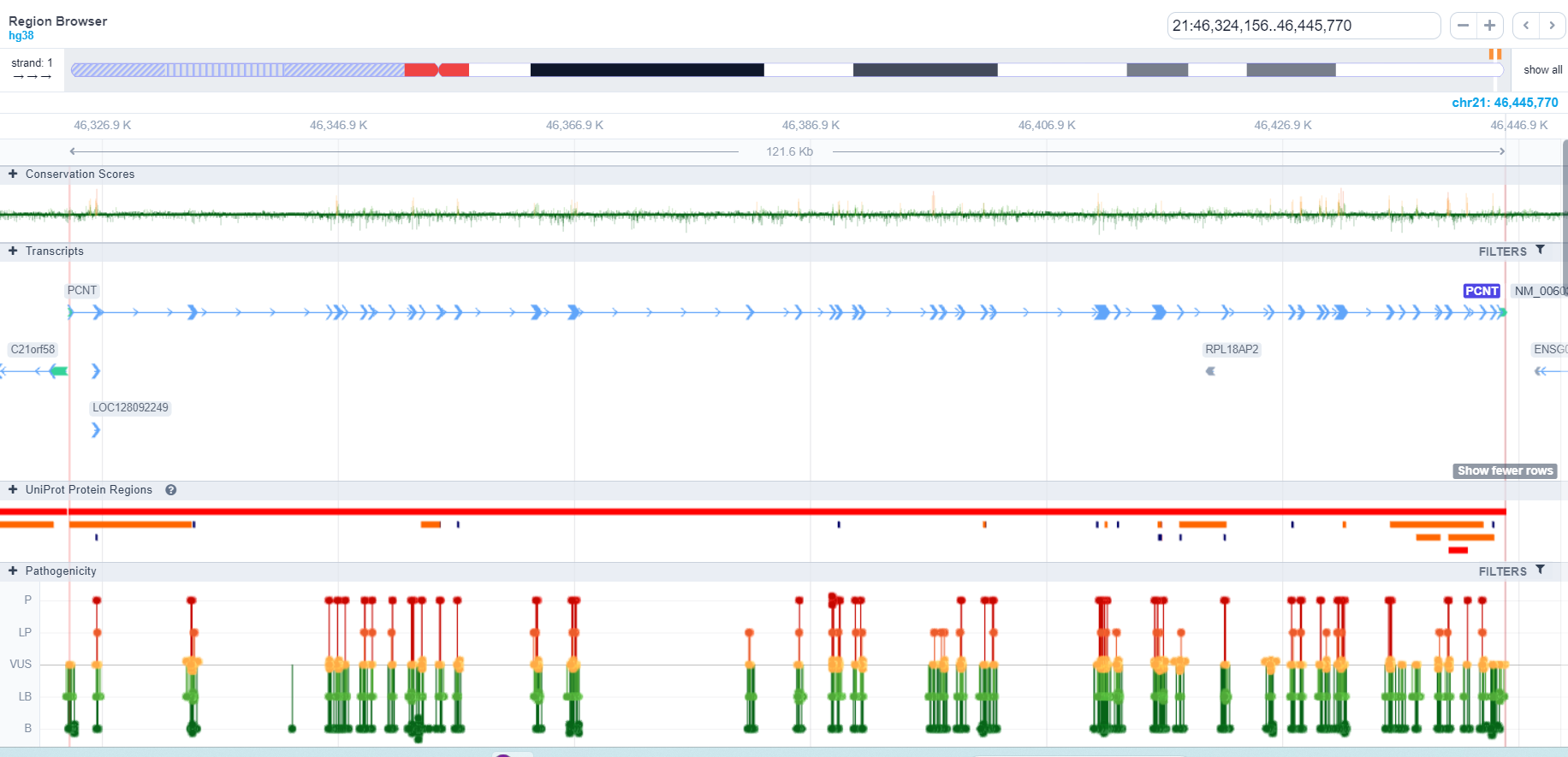


CNV777


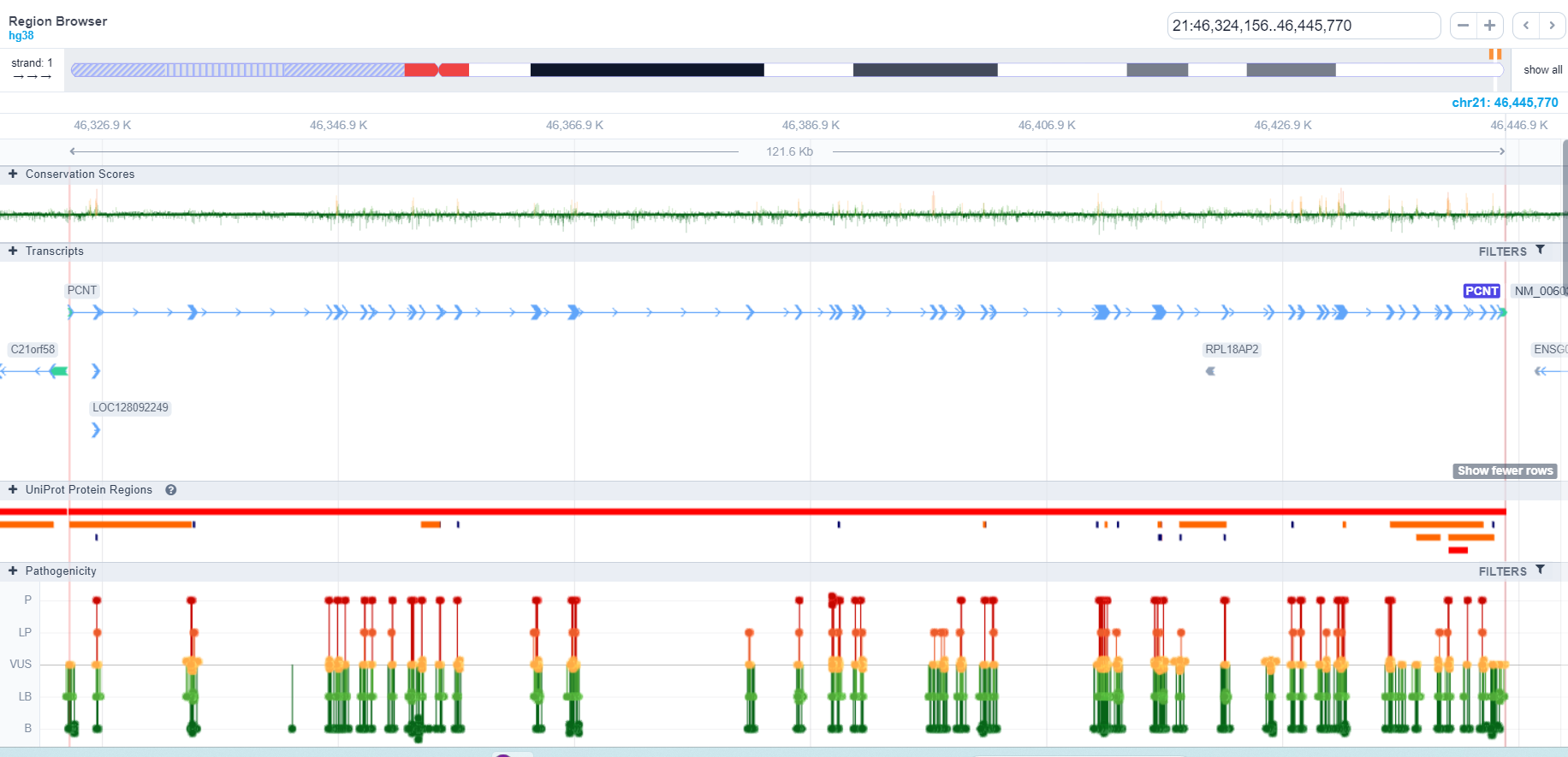


CNV1069


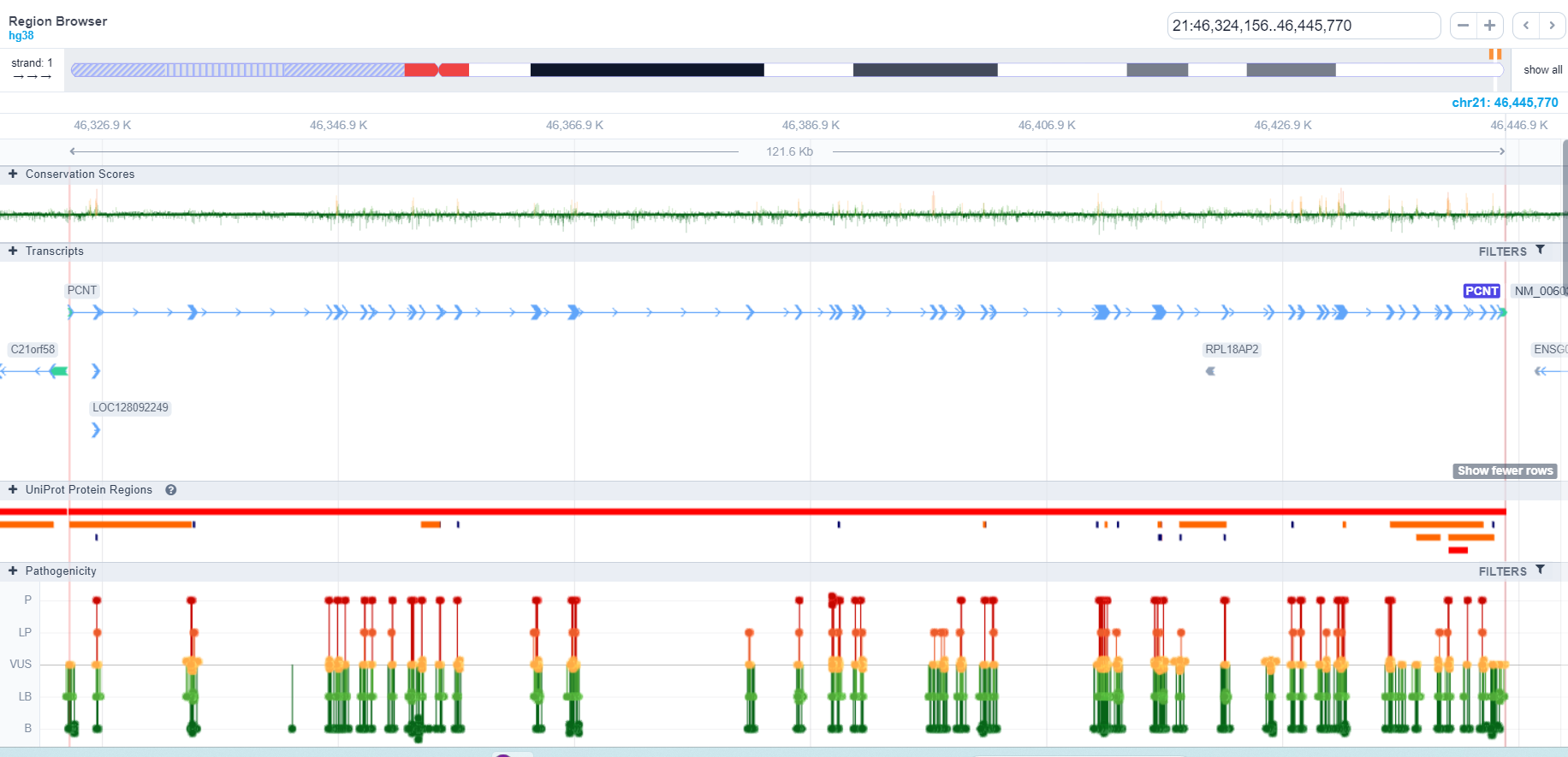


CNV3510


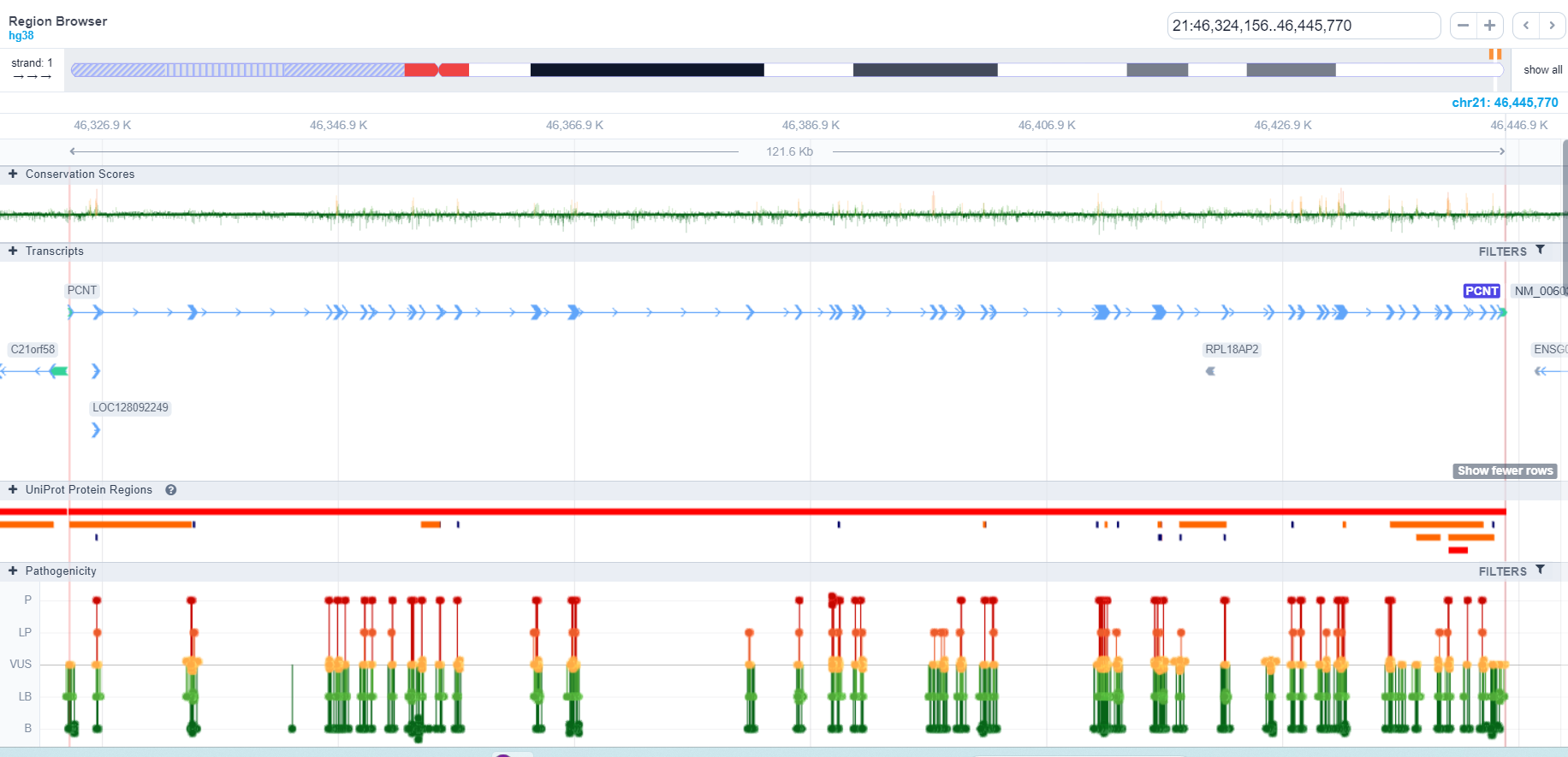


CNV3188


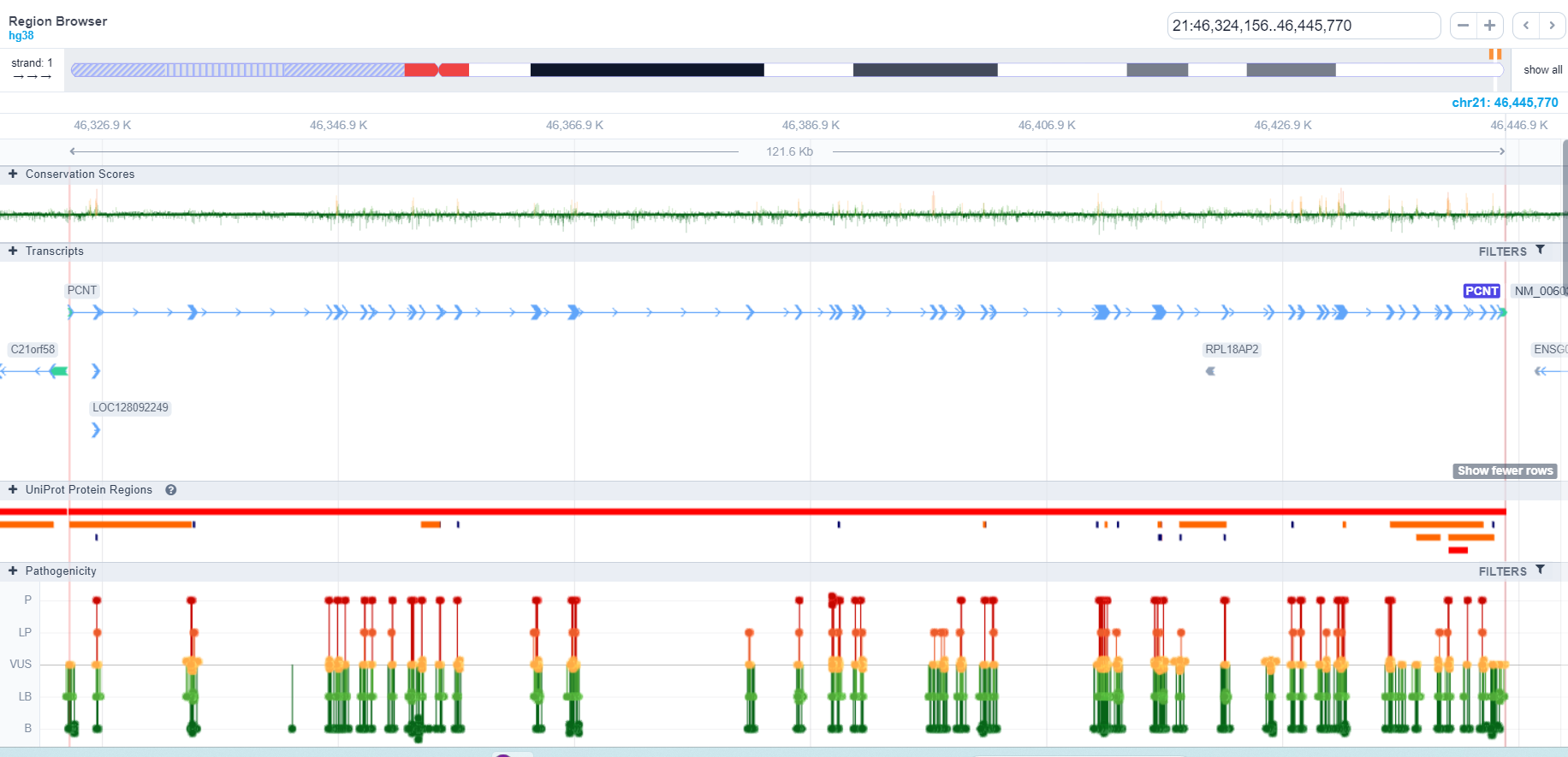


CNV3942


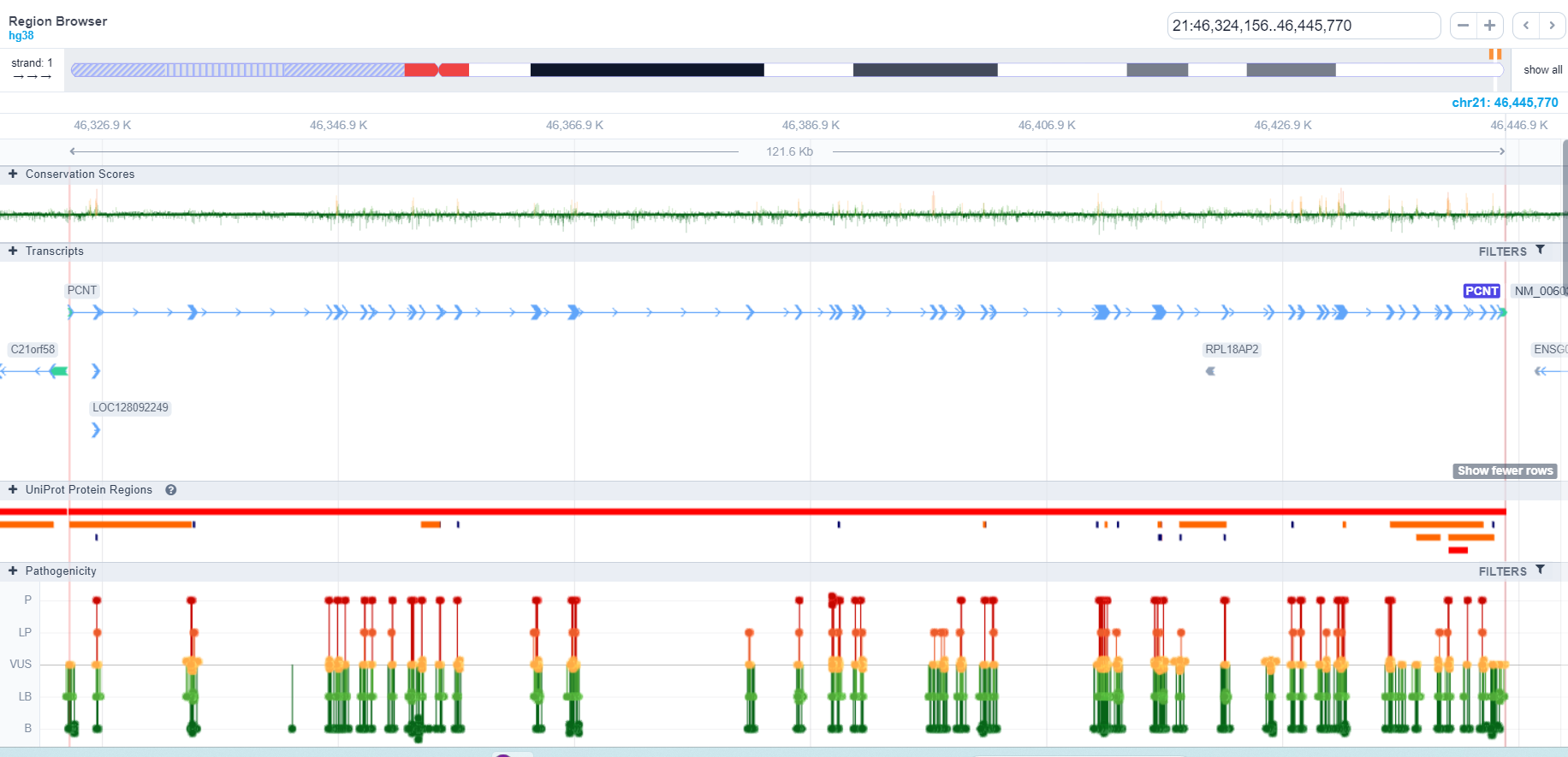

Supplement: Supplementary file 1 — Supplementary file1 (DOCX 605 KB) [file 11357_2024_1178_MOESM1_ESM.docx]
