# Supplementary material for: Genome integrity as a potential index of longevity in Ashkenazi Centenarian’s families
Source: GeroScience. 2024 May 9;46(5):4147–62. doi: 10.1007/s11357-024-01178-0 (PMC11335978; doi:10.1007/s11357-024-01178-0)
Supplement: Supplementary file 3 — Supplementary file3 (DOC 33 KB) [file 11357_2024_1178_MOESM3_ESM.doc]

**Supplement table 2.** Comparative analysis of gains, losses and length of CNVs among centenarians, progeny and control

| ***Group (No.of individuals)*** | ***CE***  ***(N=48)*** | ***Prog.***  ***(N=45)*** | ***Con***  ***(N=50)*** | ***p-value for CE vs Con.*** | ***p-value for CE vs Prog.*** | ***p-value for Prog vs Con.*** | ***p-value Among all***  ***groups*** |
| --- | --- | --- | --- | --- | --- | --- | --- |
| **Number of CN gains** | *322 ± 58 (312) | 296 ± 51 (283) | 292 ± 32 (290) | 0.0117 | 0.0146 | 0.6709 | 0.0153 |
| **Number of CN losses** | 669 ± 61 (651) | 696 ± 32 (703) | 708 ± 40 (705) | <.0001 | 0.0011 | 0.2479 | 0.0001 |
| **Total No. of CNVs** | 991 ± 58 (999) | 992 ± 45 (983) | 1000 ± 40 (1003) | 0.4469 | 0.8056 | 0.1667 | 0.  4294 |
| **Total length of CN gains (bp)** | 8927167 ± 1372225 (8985011) | 8711808 ± 806546 (8660780) | 84458120 ±  1124542 (8369250) | 0.0447 | 0.3787 | 0.139 | 0.093 |
| Total length of CN losses (bp)  Length of both gains  and losses | 8260318 ± 745270 (8191710)  17187485 ±  13891643  (16988538) | 8124418 ± 567553 (8150466)  16836225 ± 684853  (16836404) | 7970449 ± 639756 (7860302)  16416268 ± 1279085  (16122746) | 0.0333  0.0048 | 0.4129  0.1230 | 0.1691  0.018 | 0.0893  0.0068 |
| *Mean ± SD (Median) | Centenarian = CE | Progeny = Prog | Control = Con |  |  |  |  |
